# Laws of New York Establishing and Regulating the Office of Coroner

**Published:** 1873-09

**Authors:** 


### Editorial.

### Laws of New York Establishing and Regulating the Office of Coroner.

Almost every one familiar with the workings of the present Coroner system has seen how imperfect and unsatisfactory it is. It is a very ancient, important, honorable and responsible office, designed originally for the certain and early detection of crime and protection of human life. The law regulating the duty of the coroner makes it necessary to investigate the causes of death whenever there is suspicion of crime, and in violent and sudden death. The former is the true and legitimate object of the office, but there is no more reason for a Coroners inquest in cases of sudden death than in any other death.

If a man drops dead on the street, or is found dead in his bed it is no evidence of crime, indeed it is presumptive proof of innocence, such cases are rarely ever associated with guilt. Rupture of vessels in the brain, Aneurism and some diseases of the heart terminate suddenly in death, but leave no suspicion of crime, so that sudden death is no ground of inquest unless there are other circumstances calculated to awaken suspicion of guilt. When an old man turns over in his bed and dies unexpectedly to his friends, a Coroners inquest is an absurdity and often times an outrage. The cases of violent death as it is called, do not as a rule involve any criminal intent, but they might all be safely left to the discretion of these officers, if their discretion was left to govern in the premises and their salaries not dependent upon services rendered. As it is, we suffer inquest before we are fairly dead, in view of the contingency that we may possibly die, and our *ante-mortem* statement is sometimes taken when the probabilities of death are as remote as ever. The competition in the coroner business is one of the greatest objections to the present system. In Erie County we have four Coroners while in reality we do not deserve but *one.* One Coroner for Erie county with an adequate salary and he required to investigate all cases which he might deem necessary, would relieve us of nine out of ten of the Coroners inquests.

A few years since we published some complaints by a correspondent against the workings of this business and suffered Coroners inquest next day, in the verdict being charged with murder in several instances and saved from gallows by timely interference of a friendly Coroner. We do not mean to find any fault with these officers, at all; they are all very *efficient* and wholly *alive* to the true interests of the offices they hold. But we should be protected by making the office a salaried one, and thus get rid of the intolerable nuisance of having one or two Coroners appear, like Banquo's ghost, at our banquets even, ready to make *ante mortem* and *post mortem* examination whether we die of our own carelessness, or by the unfriendly hand of another, or the merciful visitation of God. This office should be held by a Physician, but he should be also a *Judge*, independent to select, impartial to decide, with full powers to investigate, receiving ample pay, and in all respects above suspicion. Some unimportant legislation was had last winter upon this subject, but nothing which reaches the root of the evil. The work done is not perhaps overpaid, but Erie County Coroner's account might be advantageously reduced to one-tenth the usual amount, with great advantage to some who die, and to nearly all who live and pay taxes annually.

